# Security Implications of AI Chatbots in Health Care

**DOI:** 10.2196/47551

**Published:** 2023-11-28

**Authors:** Jingquan Li

**Affiliations:** 1 Hofstra University Hempstead, NY United States

**Keywords:** security, privacy, chatbot, AI, artificial intelligence, health information, HIPAA, ChatGPT, computer program, natural language processing, tool, improvement, patient care, care, data security, guidelines, risk, policy

## Abstract

Artificial intelligence (AI) chatbots like ChatGPT and Google Bard are computer programs that use AI and natural language processing to understand customer questions and generate natural, fluid, dialogue-like responses to their inputs. ChatGPT, an AI chatbot created by OpenAI, has rapidly become a widely used tool on the internet. AI chatbots have the potential to improve patient care and public health. However, they are trained on massive amounts of people’s data, which may include sensitive patient data and business information. The increased use of chatbots introduces data security issues, which should be handled yet remain understudied. This paper aims to identify the most important security problems of AI chatbots and propose guidelines for protecting sensitive health information. It explores the impact of using ChatGPT in health care. It also identifies the principal security risks of ChatGPT and suggests key considerations for security risk mitigation. It concludes by discussing the policy implications of using AI chatbots in health care.

## Introduction

A chatbot is a computer program that uses artificial intelligence (AI) and natural language processing to understand customer questions and automate responses to them, simulating human conversation [1]. ChatGPT, a general-purpose chatbot created by startup OpenAI on November 30, 2022, has become a widely used tool on the internet. AI chatbots have the potential to improve patient care and public health. They can help automate routine tasks that take up unnecessary time and manpower. They can assist health care providers in providing patients with information about a condition, scheduling appointments [2], streamlining patient intake processes, and compiling patient records [3]. The chatbots can potentially act as virtual doctors or nurses to provide low-cost, around-the-clock AI-backed care. According to the US Centers for Disease Control and Prevention, 6 in 10 adults in the United States have chronic diseases, such as heart disease, stroke, diabetes, and Alzheimer disease. Under the traditional office-based, in-person medical care system, access to after-hours doctors can be very limited and costly, at times creating obstacles to accessing such health care services [3]. The chatbots can provide health education about disease prevention and management, promoting healthy behaviors and encouraging self-care [4]. For example, ChatGPT may help patients with chronic conditions in various ways. It can provide reminders for scheduling routine screenings and filling prescriptions; it can assist with other wellness matters, such as monitoring steps taken, heart rates, and sleep schedules; it can also customize nutrition plans [3].

However, the use of AI chatbots requires the collection and storage of large volumes of people’s data, which raises significant concerns about data security and privacy. The successful function of AI models relies on constant machine learning, which involves continuously feeding massive amounts of data back into the neural networks of AI chatbots. If the data used to train a chatbot include sensitive patient or business information, it becomes part of the data set used by the chatbot in future interactions. In other words, the data can be disclosed to any intended and unintended audiences and used for various purposes without authorization. Even though AI chatbots are perceived to have limited capacity, they have an enormous potential to acquire and collect new information from various data sources and capture people’s responses. The tasks of ensuring data security and confidentiality become harder as an increasing amount of data is collected and shared ever more widely on the internet.

Additionally, it will be important to consider security and privacy concerns when using AI chatbots in health care, as sensitive medical information will be involved. Once the information is exposed to scrutiny, negative consequences include privacy breaches, identity theft, digital profiling, bias and discrimination, exclusion, social embarrassment, and loss of control [5]. In the United States, covered entities, including health care providers, health plans, and health care clearinghouses, disclose protected health information (PHI) without patient authorization in violation of the 1996 HIPAA (Health Insurance Portability and Accountability Act) and can be subject to fines and other penalties. PHI refers to any information in the medical records or designated record set that can be used to identify an individual; this information was created, used, or disclosed in the course of providing a health care service, such as diagnosis or treatment [6]. However, OpenAI is a private, for-profit company whose interests and commercial imperatives do not necessarily follow the requirements of HIPAA and other regulations, such as the European Union’s General Data Protection Regulation. Therefore, the use of AI chatbots in health care can pose risks to data security and privacy.

There is an urgent need to address the security and privacy issues of AI chatbots as they become increasingly common in health care. The importance of security and privacy issues in health care is well recognized by previous research [3-12]. Although several studies have attempted to address the security and privacy issues in big data and the Internet of Things from a technological standpoint [13-17], literature specifically on policy considerations of AI chatbots in health care is quite limited. This paper addresses the gap by identifying the security risks related to AI tools in health care and proposing some policy considerations for security risk mitigation.

## AI Chatbots in Health Care

Since its launch on November 30, 2022, ChatGPT, a free AI chatbot created by OpenAI [18], has gained over a million active users [19]. It is based on the GPT-3.5 foundation model, a powerful deep learning algorithm developed by OpenAI. It has been designed to simulate human conversation and provide human-like responses through text box services and voice commands [18]. OpenAI officially released GPT-4 on March 13, 2023. GPT-4 surpasses ChatGPT in its advanced understanding and reasoning abilities and includes the ability to interact with images and longer text [20]. At present, GPT-4 is only accessible to those who have access to ChatGPT Plus, a premium service from OpenAI for which users have to pay US $20 a month.

As an AI chatbot, ChatGPT is designed to interact with humans and respond to their conversations and requests. It can be used to answer questions; write essays, poetry, or music; and compose emails and computer code. It is used by entities in a wide variety of industries, such as customer service, e-commerce, and health care. In health care, it can be used to schedule appointments, answer medical questions, and do paperwork. Dr ChatGPT [21] can potentially help create a personalized treatment for patients by considering their medical history, symptoms, social drivers, genomic data, health monitoring information, and other factors. Furthermore, it has the potential to assist drug development by identifying potential targets for new drugs and predicting the compounds that are most likely to be effective [22].

Despite its many benefits, ChatGPT also poses some data security concerns if not used correctly. ChatGPT is supported by a large language model that requires massive amounts of data to function and improve. The more data the model is trained on, the better it gets at detecting patterns, anticipating what will come next, and generating plausible text [23]. The integration of ChatGPT in health care could potentially require the collection and storage of vast quantities of PHI, which raises significant concerns about data security and privacy.

Another challenge involves the data provided to ChatGPT in the form of user prompts. When users ask the tool to answer some questions or perform tasks, they may inadvertently hand over sensitive personal and business information and put it in the public domain. For instance, a physician may input his patient’s name and medical condition, asking ChatGPT to create a letter to the patient’s insurance carrier. The patient’s personal information and medical condition, in addition to the output generated, are now part of ChatGPT’s database. This means that the chatbot can now use this information to further train the tool and incorporate it into responses to other users’ prompts.

Furthermore, as ChatGPT is applied to new functions, such as health care and customer service, it will be exposed to an increasing amount of sensitive information [23]. It will also become more challenging for people to avoid sharing their information with it. Moreover, once data are collected, they can be disclosed to both intended and unintended audiences and used for any purpose. In addition to millions of ChatGPT users all over the world, OpenAI indicates that they “share [the user’s] personal information with third parties in certain circumstances without further notice to [them], unless required by law,” according to the firm’s privacy policy [24]. OpenAI can also share personal data with law enforcement agencies if required to do so by law [24].

**Figure 1:**
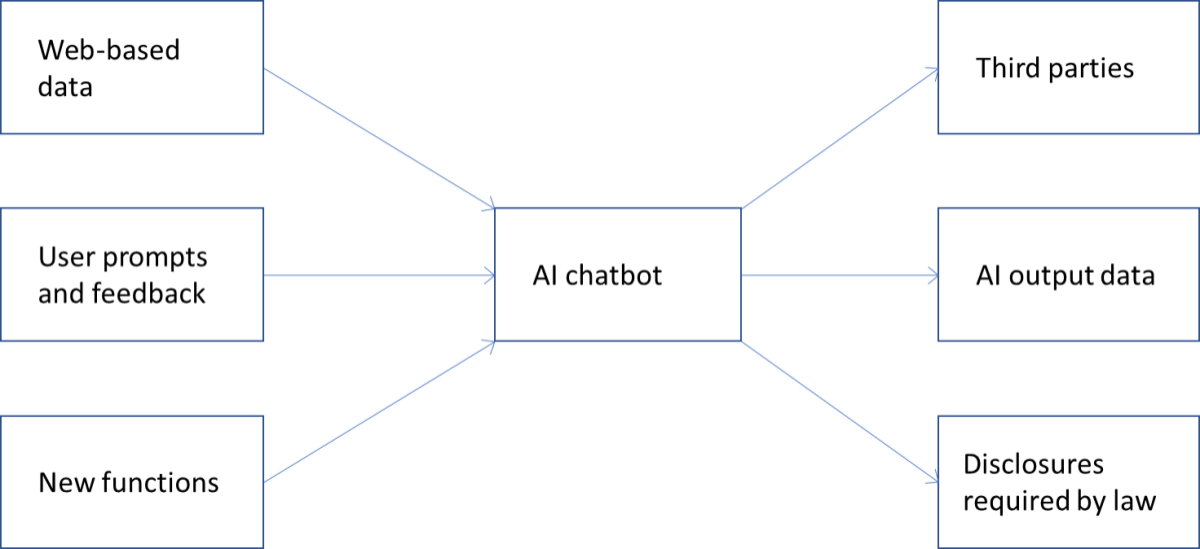
Inputs and outputs of an artificial intelligence (AI) chatbot.

ChatGPT requires massive quantities and diverse types of digital data; however, like other technologies, it is vulnerable to data breaches. An attack could feasibly jeopardize data security from the inputs, processes, and outputs of ChatGPT (Figure 1). Given personal health information is among the most private and legally protected forms of data, AI chatbots, like any other technology used in the health care industry, should be used in compliance with HIPAA. This includes ensuring the confidentiality, integrity, and availability of PHI as it is collected, stored, and shared. Since the current free version of ChatGPT does not support (nor does it intend to support) services covered under HIPAA through accessing PHI, the use of ChatGPT in health care can pose risks to data security and confidentiality.

## Security Risks

ChatGPT can have several security risks. First, the model is trained on billions of data points, which means it has access to a vast amount of people’s data without their permission [25]. This is a clear violation of data security, especially when data are sensitive and can be used to identify individuals, their family members, or their location. Moreover, the training data that OpenAI scraped from the internet can also be proprietary or copyrighted. Consequently, this security risk may apply to sensitive business data and intellectual property. For example, a health care executive may paste the institution’s confidential document into ChatGPT, asking it to review and edit the document. In fact, as an open tool, the web-based data points on which ChatGPT is trained can be used by malicious actors to launch targeted attacks.

Second, sensitive information can be leaked into the public domain when using ChatGPT. When users ask ChatGPT to answer some questions, they may inadvertently hand over sensitive patient or business information and put it in the public domain. Therefore, using ChatGPT can put users at risk of losing control of sensitive patient information, source code, and more. Potential data leakage has led companies, such as JPMorgan Chase & Co, Verizon Communications, and Amazon to limit and ban their employees from using it [26]. More importantly, the integration of ChatGPT into existing health care systems could potentially require the use and disclosure of vast amounts of PHI; however, this may cause data leaks involving a variety of unintended entities and applications. According to its privacy policy, OpenAI may disclose users’ personal information to its business partners, law enforcement officials, other third parties, and other users [24].

Third, another concern is the lack of transparency regarding the origin of the sensitive data used to train the model. It can be difficult for people to know if their data have been used to train the model. In that case, they may want to have the ability to change or erase their data from the model. This “right to be forgotten” is particularly important in cases where the information is inaccurate or misleading, which seems to be a regular occurrence with ChatGPT [25].

Fourth, phishing emails are one of the most common security threats from ChatGPT. Malicious actors can easily use ChatGPT to write convincing phishing codes and emails that appear to be from legitimate sources [27]. Given that many hackers are nonnative English speakers, automated phishing emails with effective writing could enhance their success in tricking people and organizations out of money. These emails can be used to steal personal information, such as passwords and credit card numbers.

Fifth, malware is another common security threat from ChatGPT. Malware is malicious software that can be used to steal sensitive data, hijack computers, and perform other malicious activities. ChatGPT provides less experienced and less skilled hackers with the opportunity to write accurate malware code [27]. It is bad enough that malware is already widely available. AI chatbots like ChatGPT can aid in malware development and will likely exacerbate an already risky situation by enabling virtually anyone to create harmful code themselves.

Finally, another obvious issue is the scale of the security risk. The more dependent people are on technology, the more at risk they are when a system goes down. Encrypted transmission will improve confidentiality and multifactor authentication, and access control will reduce nonauthorized access; however, a single “hack” into the system may compromise lots of sensitive information or lead the technology to provide incorrect, deliberately misleading, or biased information.

## Proposed Security Safeguards

One of the most critical considerations in implementing AI chatbots like ChatGPT is ensuring data security and privacy. This is even more important in highly regulated industries, such as health care delivery, pharmaceutical delivery, banking, and insurance, where AI tools collect client information. The lack of a robust AI security and privacy framework can result in data breaches, reputational damage, reduced consumer trust, compliance and regulatory violations, as well as heavy fines and penalties. ChatGPT, like any other technology used in the health care industry, must be used in compliance with HIPAA regulations. This includes ensuring the confidentiality, integrity, and availability of PHI as it is collected, stored, and shared.

Health care institutions that use ChatGPT should implement strict data security measures for the use and disclosure of PHI. They should conduct regular risk assessments and audits to ensure compliance with HIPAA and any applicable privacy law. There are several important security considerations that need to be considered.

First, HIPAA requirements must be met so that PHI and other sensitive data remain private and confidential. One common way to comply with HIPAA regulations is to ensure that ChatGPT is trained and improved responsibly using anonymized data [23]. Under HIPAA, there are 2 methods for deidentification: Safe Harbor and the Statistical Method [5,6,8]. Safe Harbor requires the removal of specified identifiers from the data (eg, names, dates, and numbers). The Statistical Method requires an expert, a statistician familiar with the properties of the data, to perform techniques to mask identifiers. Once PHI has been deidentified, it is no longer considered to be PHI; as such, there are no longer restrictions on its use or disclosure.

Second, prior to implementing a new technology that potentially accesses or uses PHI, covered entities should enter into a business associate agreement with the vendor of such technology [3] and ensure that appropriate provisions governing disclosure, use, and protection of such PHI as well as notification requirements in the event of a data breach are in place.

Third, organizations that combat AI chatbot security concerns should ensure solid identity and access management [28]. Organizations should have strict control over who has access to specific data sets and continuously audit how the data are accessed, as it has been the reason behind some data breaches in the past [11]. Furthermore, moving large amounts of data between systems is new to most health care organizations, which are becoming ever more sensitive to the possibility of data breaches. To secure the systems, organizations need to let the good guys in and keep the bad guys out by ensuring solid access controls and multifactor authentication as well as implementing end point security and anomaly detection techniques [29].

Fourth, security audits, which provide a means of independently verifying that ChatGPT operates according to its security and privacy policies [8], should be conducted. Although OpenAI considers safety to be a high priority, it does not provide transparency into what data sets it is using to train ChatGPT, how its algorithm and AI model work, where and how the data are stored, how the data need to be accessed and used, and how the data are shared and disclosed with unintended individuals and entities. Security audits evaluate security and privacy practices retrospectively. A chatbot cannot assure users of their security and privacy unless it enables users to request an “audit trail,” detailing when their personal information was accessed, by whom, and for what purpose [8]. Paired with proactive risk assessments, auditing results of algorithmic decision-making systems can help match foresight with hindsight, although auditing machine-learning routines is difficult and still emerging.

Fifth, another way to mitigate ChatGPT security risks is to implement strict security measures for storing and transmitting PHI, such as encryption, secure authentication protocols, and network detection and response solutions [13]. Powered by ChatGPT, malicious actors are increasingly using complicated techniques via the internet to steal personal information. Encryption technology for the transmission and storage of personal information provides enhanced security. Organizations may develop strong secure authentication protocols that combine two or more independent categories of credentials: what the user knows (password), what the user has (security token), and what the user is (a biometric characteristic, such as a fingerprint) [8]. They may also invest in things like network detection and response solutions that monitor the network 24/7 [27], reporting suspicious activities or vulnerabilities. Social engineering and phishing attacks are the most important threats to users. Sadly, there is no computer program that can protect the network from social engineering or phishing attacks. The best protection from these risks is security education and awareness.

Finally, another way to mitigate ChatGPT risks is to establish rules for how AI is used in the workspace and provide security awareness education to users. As AI technologies become increasingly sophisticated, the potential for inadvertent disclosure of sensitive information may increase. For instance, health professionals may inadvertently reveal PHI if the original data were not adequately deidentified. How many times have you unintentionally copied and pasted your personal information such as login ID and password into Google search or the address bar? As ChatGPT continues to evolve and be integrated into more aspects of our work, there are increasing risks of feeding it sensitive information, such as patient data, source code, or proprietary information, that could be collected and stored by OpenAI and retrieved later by other users. An acceptable use policy should stipulate a set of rules that a user must agree to for access to an AI tool. The policy should prevent a user from entering sensitive business or patient information into these AI tools. One effective way for users to combat the risks is by undertaking AI security awareness training [12].

These security policy considerations should inform deliberations about the security challenges and concerns of AI chatbots in health care. In principle, many of the techniques and industry best practices needed to implement and enforce these security considerations are available, if not deployed on AI platforms. This paper only provides a concise set of security safeguards and relates them to the identified security risks (Table 1). All the protections for health data are not detailed in this paper. It is important for health care institutions to have proper safeguards in place, as the use of chatbots in health care becomes increasingly common.

**Table 1:** Artificial intelligence (AI) security risks and safeguards.

Security risks	Security safeguards
Data breaches	Using anonymized data; data masking; desensitizing data; business associate agreement; identity and access management; breach notification and enforcement
Leakage to AI chatbots	Acceptable use policy; AI security awareness training; sharing the minimum amount of person-specific data to accomplish the intended purpose (when in doubt, err on the side of providing less data)
Lack of transparency	Security audits; proactive risk assessments; auditing monitoring of algorithm decision-making; transparency of data handling practices
Phishing or social engineering attacks, malware, and interface spoofing	Technical barriers, such as encryption, secure authentication protocols, as well as network detection and response solutions; organizational measures, such as user security awareness training; implementing end point security and anomaly detection techniques
Scale of security risk	Implementing strict security measures for storing and transmitting protected health information, such as encryption and access controls; business associate agreement; restricting the uses and disclosures of protected health information to the minimum amount necessary to achieve the purpose for which it is being used, requested, or disclosed

## Conclusions

AI chatbots like ChatGPT have the potential to improve patient care and public health. However, some key security implications must be considered before ChatGPT can be integrated into AI-driven health care applications. First, HIPAA requirements must be met so that electronic PHI and other sensitive data remain private and secure. Users must ensure the anonymity of the data fed into the chatbot and the implementation of appropriate measures for data security. Second, before entrusting an AI chatbot with sensitive data, health care organizations should establish a business associate agreement to hold the vendor of such technology to the same rigorous data protection standards. Third, it is important to implement strict security safeguards for storing and transmitting PHI, such as encryption, identity and access management, security auditing, network detection and response, and security intelligence.

Furthermore, it is important to engage users in protecting sensitive patient and business information. For many people, it might be common sense not to feed ChatGPT PHI, source code, or proprietary information; however, some people might not fully understand the risks attached to it. As users of a growing number of AI technologies provided by private, for-profit companies, we should be extremely careful about what information we share with such tools.

AI chatbots need lots of data to train their algorithms, and some top-rated chatbots like ChatGPT will not work well without constantly collecting new data to improve the algorithms. This implies that AI chatbots will continue to compromise data security and privacy. Nevertheless, there are many ways to improve the collection, use, and disclosure of data, including overall data management and the algorithms themselves. Future studies are required to explore data desensitization methods, secure data management, and privacy-preserving computation techniques in web-based AI-driven health care applications.

## Abbreviations

AI: artificial intelligence
HIPAA: Health Insurance Portability and Accountability Act
HPI: protected health information
